# The Roles of Enzymes as Dietary Additives in Ruminant Diets: A Meta-Analysis

**DOI:** 10.3390/ani15243631

**Published:** 2025-12-17

**Authors:** Diky Ramdani, Ririn Siti Rahmatillah, Yulianri Rizki Yanza, Anuraga Jayanegara, Nasrul Wathoni, Abdul Shakoor Chaudhry

**Affiliations:** 1 Department of Animal Production, Faculty of Animal Husbandry, Universitas Padjadjaran, Jatinangor Campus, Sumedang 45363, Indonesia; ririn17003@mail.unpad.ac.id; 2 Department of Animal Nutrition and Feed Technology, Faculty of Animal Husbandry, Universitas Padjadjaran, Jatinangor Campus, Sumedang 45363, Indonesia; y.r.yanza23@unpad.ac.id; 3 Department of Animal Nutrition and Feed Technology, Faculty of Animal Science, IPB University, Bogor 16680, Indonesia; anuragaja@apps.ipb.ac.id; 4 Department of Pharmaceutics and Pharmaceutical Technology, Faculty of Pharmacy, Universitas Padjadjaran, Jatinangor Campus, Sumedang 45363, Indonesia; nasrul@unpad.ac.id; 5 School of Natural and Environmental Sciences, Newcastle University, Newcastle upon Tyne NE1 7RU, UK; abdul.chaudhry@newcastle.ac.uk

**Keywords:** additives, enzymes, in vitro, in vivo, ruminants

## Abstract

Understanding the function of enzymes before their use as additives in ruminant diets is essential for achieving sustainable and efficient agricultural practices. Ruminants such as cattle, sheep, and goats are vital for global food production because of their ability to convert fibrous plant materials into high-quality proteins through enteric fermentation. Various datasets were carefully selected from four scientific databases: Science Direct, Scopus, PubMed, and Google Scholar. The rigorous Preferred Reporting Items for Systematic Review and Meta-Analysis (PRISMA) protocol was employed to ensure the eligibility of the selected articles used in the analysis. A systematic review showed that the administration of various types of enzymes can increase dry (DMD) and organic (OMD) matter, neutral (NDFD), and acid (ADFD) detergent fibre, and crude protein (CPD) digestibility in both in vitro and in vivo tests in individual studies. However, the pooled meta-analysis indicated that their overall effect on CPD was not significant (*p* > 0.05). The OpenMEE approach was used to calculate the effect size (Hedges’ g) for each experimental unit for various parameters. Across enzyme types and doses in the meta-analysis, the administration of enzymes did not have any significant effect (*p* > 0.05) on DMI, OMI, and CPD, but it did have a significant effect (*p* < 0.05) on DMD, OMD, ADFD, NDFD, pH and gas production at 24, 48, and 72 h, as assessed by in vitro experiments. Ruminant in vivo studies indicated that the administration of enzymes has significant impacts (*p* < 0.05) on digestibility parameters (DMD, OMD, NDFD, ADFD), milk production, milk lactose content, acetate, and propionate, but it had non-significant impacts on milk protein and rumen total volatile fatty acids and acetate: propionate ratio.

## Simple Summary

Ruminants like cattle, sheep, and goats play a key role in global food production by converting fibrous plants into valuable protein. This study reviewed and analyzed published research to understand how enzyme additives affect ruminant feed digestibility and productivity. Using a systematic review and meta-analysis approach, the results showed that enzyme supplementation improved the digestibility of dry matter, organic matter, and fibre components in both laboratory and animal trials. It also enhanced milk yield and milk lactose in dairy animals. However, enzymes did not significantly affect feed intake, crude protein digestibility, or rumen fermentation balance. These findings suggest that enzyme additives can not only enhance nutrient utilization and productivity in ruminants, but also contribute to more efficient and sustainable livestock production.

## 1. Introduction

Optimization of ruminant nutrition is a pivotal pursuit for sustainable and efficient agricultural practices in the ever-evolving landscape of animal husbandry and livestock management. Ruminants, such as cattle, sheep, and goats, play a crucial role in global food production by converting fibrous plant materials into high-quality proteins, and other essential nutrients through enteric fermentation [1]. However, challenges such as suboptimal feed utilization and digestive inefficiencies persist, affecting animal health, productivity, and environmental sustainability.

Ruminants naturally contain microflora and enzymes that aid in the digestive processes [2]. The important role of enzymes in the digestive system of ruminants has attracted manufacturers to develop both natural and synthetic enzyme products as feed additives. These feed additives contain enzymes that are produced using batch fermentation processes, in which the enzymes are separated from the fermentation residues and organisms after microbial fermentation is completed [3].

Despite the growing availability of enzyme-based feed additives, the current understanding of their efficacy across ruminant species, production stages, and feeding backgrounds remains fragmented. The objective of this study was to systematically investigate the effect of various enzymes, including amylases, proteases, lipases, and cellulases, as exogenous feed additives in ruminant diets. By integrating a systematic review with meta-analysis, this study uniquely contributes to the field by providing a comprehensive data-driven assessment of enzyme supplementation, addressing gaps in the literature, and offering new insights into the synergistic potential of enzyme combinations. This study aimed to quantify the magnitude of the effects of enzyme supplementation on nutrient digestibility and animal performance, and to test whether these effects differ across enzyme classes, ruminant species, and basal diet types. Also, to enhance the understanding of enzyme roles in optimizing ruminant nutrition, ultimately guiding more sustainable and efficient livestock management practices.

## 2. Materials and Methods

**Figure 1:**
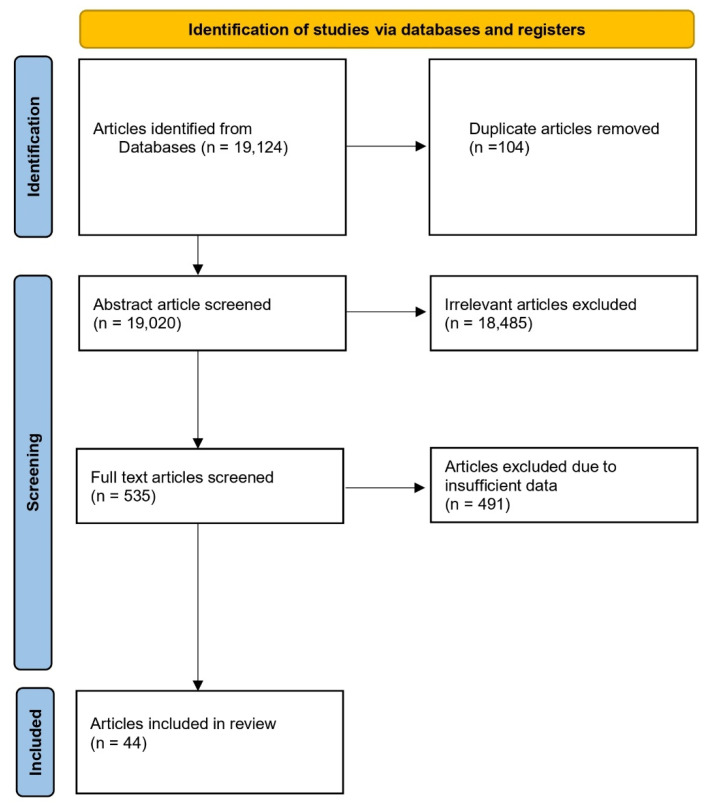
The Preferred Reporting Items for Systematic Review and Meta-Analysis (PRISMA) protocol to select eligible articles [4].

All peer-reviewed articles were selected to ensure that their quality and relevance did meet the inclusion criteria for a systematic review. The first step was to check the database of articles by using keywords “Enzyme AND Ruminant AND (Cattle OR Goat OR Sheep)” from Science Direct, Scopus, and Pub Med in the reference manager software (Mendeley Desktop 1.19.8) for duplication potential. About 19,124 articles screened and 19,020 documents were not duplicated. The next step of selection was based on the relevance of each title, indexed peer-reviewed journals, and the data availability. In this stage, only articles discussing dietary enzyme inputs were included. Articles that examined enzymes as outputs or solely explored enzyme mechanisms without presenting them as input data were excluded from the criteria. Here, only 44 articles were used. The search and article collection were conducted in two stages during 2023–2024, without applying a specific publication year range; therefore, older studies were also eligible as long as they met the inclusion criteria. Additionally, explicit inclusion and exclusion criteria were applied at each screening level, including relevance to dietary enzyme supplementation in ruminants, availability of quantitative data suitable for meta-analysis, and publication in indexed peer-reviewed journals. Reasons for exclusion included non-relevant study focus, insufficient data, review-type articles, or studies evaluating enzymes as outputs rather than inputs. All selection processes for the eligibility of the above selected articles were performed following the Preferred Reporting Items for Systematic Review and Meta-Analysis (PRISMA, Figure 1) protocol [4,5].

Meta-analysis was conducted by employing the OpenMEE software (http://www.cebm.brown.edu/openmee/index.html, accessed on 1 June 2024) for meta-analysis in biology and ecology [6]. Means of the experimental units (control vs. treatment) were set as continuous outcome data. The data were set in a comma-separated value (CSV) file and then submitted to the software. The effect size of each experimental unit was calculated for each outcome variable with Hedges’ g. The random-effects models were used where the data were displayed as standardized mean differences (SMD) between the control and treatment using the following Formula (1):

G ≅ d × (1 − 3/(4(n1 + n2) − 9))(1)

where n is the sample size of the control and treatment groups [7]. The output was presented in a forest plot table following a random-effects model at 95% confidence intervals (CIs). Hedges’ g was selected since it was widely recognized to have strong analytical power when dealing with a relatively small sample size [8]. Hedges’ g outcome estimates as a positive value with *p* ≤ 0.05 indicates a significantly higher treatment receiving enzyme supplementations compared to control. The heterogeneity index (I2) was calculated using the DerSi-monian and the Laird test (Q-statistic) at a significance level of *p* ≤ 0.05. The degree of heterogeneity was categorized as no heterogeneity (0 < I2 ≤ 25%), low (25% < I2 ≤ 50%), moderate (50% < I2 ≤ 75%), and high (I2 > 75%) [7]. The output is presented in a forest plot table following a random-effects model at 95% confidence intervals (CIs). Hedges’ g was selected because it is widely recognized to have strong analytical power when dealing with a relatively small sample size [8].

## 3. Results and Discussion

Enzymes are biological catalysts that stimulate complex biochemical reactions at temperatures relevant to living organisms and the environments in which they exist [9]. The proteinaceous nature of these molecules allows for a myriad of three-dimensional structures that will accommodate different substrate specificities and respond to the presence of other ‘‘regulatory’’ molecules such as changes in ionic environment, pH, temperature, and hydrophobicity. These interactions often result in alteration of the conformation of the enzyme protein, which in turn affects the binding of substrates, activators, inhibitors, and cofactors, or the efficiency of catalytic activity [10]. In addition to the mechanisms for controlling enzyme activity, the actual amount of enzyme protein in a cell can be controlled via a balance of synthesis and degradation, thus allowing for many tiers of regulatory control [11].

Ruminants typically consume diets containing relatively high amounts of forage, which contains cell wall fractions containing complex compounds such as β-mannans [12]. They are typically known for their structural resistance to solubility, leading to high viscosity in various feeds and exhibiting anti-nutritional properties in animal diets [13]. The digestion of plant cell walls in fibrous feeds by ruminants is possible mainly because of the enzymes produced by ruminal bacteria, protozoa, and fungi. Several studies have focused on improving the degradation of fibrous feeds in ruminants using feed additives, ionophores, directly fed microbes, and cell wall-degrading enzymes, or by using exogenous fibre-degrading enzymes to stimulate rumen digestive microorganism activities [14]. Fibrous feeds have high cellulose and hemicellulose concentrations that can create a complex of structural carbohydrates and lignin to reduce the digestibility of carbohydrates and the efficient utilization of forage by ruminants [15].

Dietary enzyme products contain concentrated enzyme activities but do not contain microbial cells. They are produced using a batch fermentation process, in which the enzymes are separated from the fermentation residues and source organism once the fermentation process is completed. The types and activities of enzymes produced can vary widely depending on the microbial strains, growth substrates, and culture conditions [16]. Most enzyme products for ruminants are manufactured mainly for their polysaccharide activities, but they also contain an array of secondary enzymes, such as glucosidase, protease, and amylase [3].

Exogenous dietary enzymes have the potential to improve feed utilization and productivity in ruminants [17], but their efficacy depends on many factors such as feed type, enzyme product, application level, and method [18]. Increased enzymatic activity is supposedly caused by synergism between rumen microbes and exogenous enzymes [18], and is possibly mediated by changes in bacterial numbers. Enhanced rumen bacterial numbers and population changes due to dietary enzyme supplementation have been reported previously [19,20]. Furthermore, the rumen fraction and sampling time are also known to influence the microbial population, which, therefore, influence the results of enzyme supplementation studies. The effects of exogenous enzymes can be categorized by their mode of action as pre-consumptive (acting on the feed), ruminal, or post-ruminal [18]. Exogenous enzymes can be defined based on their activities as amylases, cellulases, β-glucanases, hemicelluloses, xylanases, pectinases, and proteases [17].

Table 1 shows that the administration of various enzyme types results in mixed outcomes, with several studies reporting increases in DMD, OMD, NDFD, ADFD, and CPD in both in vitro and in vivo tests. In the in vivo experiments, it is known that administration of enzymes can increase DMI and milk production (kg/d), although in several studies, it can reduce milk fat, milk protein, and milk lactose. In contrast, in several in vitro tests, the administration of enzymes can reduce gas production (GP) and rumen pH. Variations in gas production responses to enzyme supplementation reported in the literature can be attributed to differences in enzyme type, dosage, substrate composition, and microbial adaptation. Some studies observed increased gas production [15] because these enzymes enhance the hydrolysis of fibrous carbohydrates, providing more fermentable substrates for rumen microbes. In contrast, other studies [21] reported decreased or unchanged gas production with cellulase and xylanase, as this study argues that the high doses of additives (*Salix babylonica* extract) in high-concentrate diets can affect ruminal fermentation and in vitro gas production parameters.

Table 2 shows the results of the meta-analysis of the impact of enzymes as dietary additives in ruminant diets based on in vitro studies. The administration of enzymes as feed additives did not have a significant effect (*p* > 0.05) on DMI, OMI, and CPD but had a significant effect on pH, DMD, ADFD and NDFD (*p* < 0.05), GP 24 (*p* < 0.01), GP 48, and GP 72 h (*p* < 0.001). This lack of a significant effect on these parameters suggests that enzyme supplementation may not enhance the overall nutritional intake and digestibility of feeds under in vitro conditions.

Previous studies reported that enzyme supplementation did not improve DMI, OMI, DMD, OMD, CPD, ADFD, and NDFD. For example, Beauchemin et al. (2003) [17] and Eun and Beauchemin (2007) [24] noted that enzyme supplementation did not significantly affect the digestibility of dry matter and fibre in ruminant diets. These findings suggest a consistent pattern in which adding enzymes to ruminant diets does not enhance these specific nutritional metrics in in vitro studies, likely because of the complex interactions between enzymes and feed components that are not fully replicated in vitro [59].

In contrast, the significant effects of enzyme supplementation on pH, GP 24, GP 48, and GP 72 h suggest that dietary enzymes may play a role in altering the fermentation characteristics of different feeds. The significant changes in pH and GP indicate that enzymes can modify the fermentation environment, potentially leading to more efficient microbial activity and fermentation processes.

The results align with the previous study reported by Nsereko et al. (2000) [60] and Colombatto et al. (2003) [61], who observed that enzyme supplementation could alter fermentation parameters, such as pH and GP. This modification in pH could be attributed to the breakdown of complex carbohydrates by enzymes, leading to the production of volatile fatty acids and subsequent changes in the fermentation environment. Similarly, the increase in GP might be due to the enhanced microbial activity and fermentation efficiency in the presence of enzyme supplementation.

This highlights the potential of enzymes to influence the microbial ecosystem and fermentation processes in the rumen, which could improve feed efficiency and overall animal performance in vivo. Further research is needed to explore the mechanisms underlying these effects and determine the practical applications of enzyme supplementation in ruminant nutrition.

Table 3 indicated that the administration of enzymes as feed additives caused significant differences in the digestibility parameters. Specifically, significant effects (*p* < 0.05) were observed on DMD, OMD, ADFD, or NDFD. Among milk production parameters, enzyme administration had a significant impact on milk production (*p* < 0.001) and lactose content (*p* < 0.05), but it did not have a significant impact on protein, and fat content of milk. Based on fermentation outputs, enzyme supplementation had a significant influence on acetate and propionate (*p* < 0.05), but did not show significant impact on total VFA and the acetate:propionate ratio (A:P).

Some studies have reported improvements in DMD with enzyme supplementation, particularly in diets containing high levels of indigestible components [62]. However, the current meta-analysis indicated that these improvements were not consistent across different studies, potentially due to variations in diet composition, enzyme type, and animal species.

Although enzymes are hypothesized to break down complex organic materials and enhance nutrient availability, the overall impact appears to be negligible when averaged across multiple studies [63]. This suggests that factors such as enzyme activity levels, feed processing methods, and animal adaptation to enzyme supplements may play crucial roles in determining the outcomes of such interventions.

**Figure 2:**
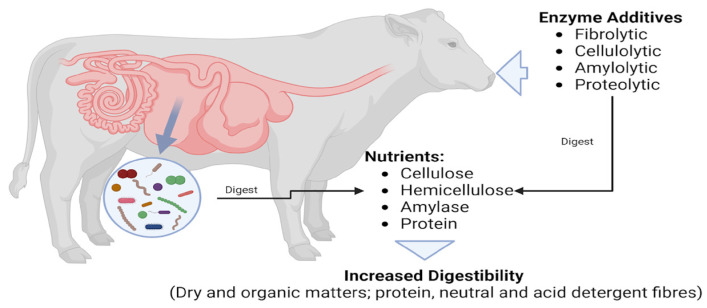
Graphical abstract of the role of enzymes in the ruminant system.

Enzymes, particularly fibrolytic enzymes, are expected to degrade fibre components, thereby improving the digestibility of acid and neutral detergent fibres (Figure 2). However, the meta-analysis findings imply that such benefits are not universally observed, highlighting the need for further research to identify the conditions under which enzyme supplementation can effectively enhance fibre digestibility [64].

In addition to examining digestibility parameters, this meta-analysis also explored the effects of enzyme administration on milk production parameters. Enzymes are believed to improve the overall efficiency of nutrient utilization, leading to greater energy availability for milk synthesis [65]. This enhanced nutrient absorption can contribute to increased milk yield, as observed in this meta-analysis.

Previous research has shown varying results regarding the influence of enzyme supplementation on milk composition. Some studies have reported marginal increases in milk protein and fat content, whereas others have found no significant changes [66]. The current meta-analysis suggests that enzyme supplementation can boost overall milk production, but it does not necessarily alter the protein and fat components of milk in a consistent manner.

The unchanged levels of protein, and fat may be attributed to the specific roles of these macronutrients in milk synthesis. Lactose synthesis is primarily regulated by inherent biochemical pathways in the mammary gland, which may not be directly influenced by dietary enzyme supplementation [67]. Similarly, milk protein and fat synthesis are complex processes influenced by multiple factors, including genetic, physiological, and nutritional factors, which may not be significantly affected by enzyme supplementation alone.

VFAs are key end-products of microbial fermentation of carbohydrates in the rumen, and their increased production implies enhanced breakdown and utilization of feed components [68]. This enhancement can lead to improved energy availability for animals, thereby supporting better overall performance.

Specifically, the significant impact on acetate and propionate levels is noteworthy. Acetate is primarily produced by the fermentation of fibrous carbohydrates, whereas propionate is mainly derived from the fermentation of non-fibrous carbohydrates. The observed increase in both acetate and propionate levels indicates that enzyme supplementation may improve the digestion of a wide range of carbohydrates, contributing to a more efficient rumen fermentation process [69].

A significant change in the acetate-to-propionate ratio is also of particular interest. This ratio is an important indicator of the balance between fibre and starch fermentation in the rumen. A lower acetate to propionate ratio is often associated with improved feed efficiency and energy utilization, as propionate is a more efficient precursor for glucose synthesis in ruminants [70]. The alteration in this ratio due to enzyme supplementation suggests a shift towards more efficient carbohydrate utilization and energy production.

These findings align with previous research highlighting the potential of enzyme additives to modulate rumen fermentation patterns. By improving the breakdown of both fibrous and non-fibrous carbohydrates, enzymes can enhance VFA production, thereby supporting better nutrient utilization and animal performance [71]. However, the specific mechanisms by which different types of enzymes influence VFA production and the resultant effects on animal health and productivity require further exploration. In this paper, the available studies did not consistently report enzyme classes or mechanistic variables, such as fibre-degrading on starch-degrading enzyme activities, limiting our ability to distinguish the mechanistic pathways underlying the observed changes in VFA patterns. As a result, the findings mainly represent overall effects of enzyme supplementation rather than responses to specific enzyme types and amounts.

## 4. Conclusions

While systematic reviews suggest that enzyme administration can enhance digestibility metrics such as dry matter, organic matter, neutral detergent fibre, acid detergent fibre, and crude protein in both in vitro and in vivo tests, meta-analyses have revealed mixed results. Specifically, the enzymes significantly affect these digestibility parameters across different types and doses. However, non-significant effects were observed on fermentation characteristics, including in vitro pH and gas production, in vivo milk protein, milk fat, and total volatile fatty acid profiles. These findings highlight that enzymes may improve digestibility, as they can influence fermentation processes and productivity in ruminants. These findings suggest that the main advantage of enzyme supplementation is the improvement of nutrient degradation, rather than consistent changes in fermentation end-products. The results also show that higher digestibility does not always lead to predictable shifts in rumen fermentation or animal productivity, reflecting variability in enzyme responses across studies. In addition, limited and inconsistent reporting of enzyme types and mechanistic information in the literature restricts a clearer interpretation of their specific modes of action. Future research should therefore provide better descriptions of enzyme classifications, targeted substrates, and mechanistic parameters to identify the conditions under which enzymes exert the greatest benefit on rumen function and animal performance.

## Figures and Tables

**Table 1:** The effect of enzymes as dietary additives in ruminant diets on many in vitro and in vivo parameters.

No.	References	Enzyme Type	Object	Test System	Response Variable
1	[22]	Xylanase and Cellulase	Dairy Cows	In vivo	↑DMI, ↑OMI, ↑DMD, ↑OMD, ↑NDFD, ↓Milk Fat, ↑Milk Protein, ↑Milk lactose, ↓milk kg/d
2	[23]	Sorghum specific enzyme	Dairy Cows	In vivo	↑DMI, ↓Milk Fat, ↑Milk Protein, ↑Milk lactose, ↑milk kg/d
3	[24]	Amylolytic enzyme	Dairy Cows	In vitro and in vivo	↑DMI, ↑DMD, ↑CPD, ↑ADFD, ↑NDFD, ↑Milk Fat, ↑Milk Protein, ↓Milk lactose, ↑milk kg/d
4	[25]	Proteolytic enzyme	Dairy Cows	In vitro and in vivo	↑OMI, ↑DMD, ↑OMD, ↑pH, ↓Milk Fat, ↑Milk Protein, ↑Milk lactose, ↑milk kg/d, ↓acetate, ↑propionate, ↓total VFA, A:P
5	[15]	Enzyme preparation mixture (ENZ)	Brown Swiss cows	In vitro	↑DMD, ↑OMD, ↓pH, ↑GP12, ↑GP24, ↑GP48, ↑GP72
6	[26]	Fibrolytic enzyme		In vitro	↑DMD, ↑ADFD, ↑NDFD, ↓pH
7	[27]	Cellulase and xylanase	Sheep	In vitro	↓DMD, ↓ADFD, ↓NDFD
8	[28]	Exogenous anaerobic bacterial enzymes	Sheep	In vitro	↑DMD, ↑OMD, ↑CPD, ↑ADFD, ↑NDFD
9	[21]	Cellulase and xylanase	Rumen	In vitro	↑DMD, ↓OMD, ↓pH
10	[29]	Exogenous fibrolytic enzyme	Cows	In vitro	↓pH
11	[30]	lasalocid	goat	In vitro	↑DMD, ↑OMD, ↑pH
12	[31]	Proteolytic enzymes	Cow	In vitro	↑DMD, ↑OMD, ↑ADFD, ↑NDFD
13	[32]	Cellulase and xylanase	Rumen	In vitro	↓GP12, ↓GP24, ↓GP48, ↓GP72
14	[33]	lignocellulose	Cow	In vitro, In vivo	↑DMD, Total VFA, ↑Acetate, ↑Propionate, A:P ↓pH, ↑DMD, ↑OMD, ↑NDFD, ↑ADFD, ↑Milk Production, ↓Fat, ↓Protein, ↑Lactose
15	[34]	Xylanase-rich exogenous enzyme	Dairy cows	In vivo	↓Milk Fat, ↓Milk Protein, ↓Milk lactose, ↑milk kg/d, ↓DMD
16	[35]	Β-manase	Dairy cows	In vivo	↓Milk Fat, ~Milk Protein, ~Milk lactose, ~milk kg/d, ↓ADF, ↓NDF, ↓OMD, ↑DMD
17	[36]	Enzyme extract from *Aspergillus oryzae* & *Aspergillus niger*	Dairy cows	In vivo	↓Milk Fat, ↑Milk Protein, ↓Milk lactose, ↑milk kg/d, ↑ADF, ↓NDF, ↓OMD, ↑total VFA, ↓DMD
18	[12]	Exogenous β-manase	Dairy cows	In vivo	↑Milk Fat, ↓Milk Protein, ↑Milk lactose, ↓milk kg/d
19	[37]	Fibrolytic and amylolytic enzymes	Dairy cows	In vivo	↓Milk Fat, ↓Milk Protein, ↓Milk lactose, ↓milk kg/d, acetate, propionate, total VFA, A:P
20	[38]	Exogenous enzyme from *Aspergillus* sp.	Dairy cows	In vivo	↓Milk Fat, ↑Milk lactose, ↑milk kg/d
21	[39]	Amylase and sucrose	Dairy cows	In vivo	↑Milk Fat, ↓Milk lactose, ↑milk kg/d
22	[40]	Exogenous phytase	Dairy cows	In vivo	↓Milk Fat, ↓Milk Protein, ↑milk kg/d
23	[41]	Fibrolytic enzyme	Dairy cows	In vivo	↓Milk Fat, ↓Milk Protein, ↑Milk lactose, ↑milk kg/d
24	[42]	Cellulase and xylanase	Dairy cows	In vivo	↑Milk Fat, ↑Milk Protein, ↑Milk lactose, ↑milk kg/d, ↓acetate, ↑propionate, ↓total VFA, ↓A:P
25	[14]	Fibrolytic enzyme	Dairy cows	In vivo	↑Milk Fat, ↑Milk Protein, ↑milk kd/d
26	[43]	Cellulase and xylanase	Dairy cows	In vivo	↑Milk Fat, ↑Milk Protein, ↓Milk lactose, ↑milk kg/d
27	[17]	Non-Starch Polysaccharidedase enzyme	Dairy cows	In vivo	↑Milk Fat, ↑Milk Protein, ↓Milk lactose, ↓milk kg/d, ↑acetate, ↓propionate, ↑total VFA, ↑A:P
28	[19]	Fibrolytic enzyme	Dairy cows	In vivo	↓Milk Fat, ↓Milk Protein, ↓Milk lactose, ↑milk kg/d
29	[44]	α-amylase	Beef cattle	In vivo	↓Acetate, ↑propionate, ↓A:P
30	[20]	Fibrolytic enzyme	Dairy cows	In vivo	↓Acetate, ↑propionate, A:P
31	[45]	Fibrolytic enzyme	Dairy cows	In vivo	↑Acetate, ↑propionate, ↑total VFA, ↓A:P
32	[46]	Xylanase and cellulase	Dairy cows	In vivo	↓Acetate, ↑propionate, ↑total VFA, ↓A:P
33	[47]	Xylanase and cellulase	Sheep	In vivo	↑ADF, ↑NDF, ↑OMD, ↑DMD
34	[48]	Enzyme extract from *Aspergillus oryzae* & *Aspergillus niger*	Dairy cows	In vivo	~ADF, ~NDF
35	[49]	exogenous enzymes (Amylase, Protease, Cellulase, Xylanase, Beta glucanase)	Cow	In vivo	↑DMD, ↓OMD, ↑NDFD, ↑ADFD
36	[50]	exogenous fibrolytic enzymes	Sheep	In vivo	↓DMD, ↓OMD, ↑NDFD
37	[51]	α-amylase	Cow	In vivo	~DMD, ~OMD, ↑NDFD, CPD, ↑Milk Production, ↑Lactose, ↓Protein, ↓Fat
38	[52]	fibrolytic enzyme cocktail	Goat	In vivo	↑DMD, ↑OMD, ↑NDFD, ↑ADFD, ↓Total VFA, ~Acetate, ↑Propionate, ↓A:P
39	[53]	amylase	Cow	In vivo	↑DMD, ↑OMD, ↑NDFD, ↑ADFD, ↓Milk Production, ↑Fat, ↓Protein, ↑Lactose, ↓Total VFA, ↑Acetate, ↓Propionate, ↑A:P
40	[54]	α-amylase	Cow	In vivo	↑DMD, ↑NDFD, ↑Milk Production, ~Lactose, ↓Protein, ↓Fat
41	[55]	coated folic acid & coated riboflavin	Cow	In vivo	↑DMD, ↑OMD, ↑NDFD, ↑ADFD, ↑Total VFA, ↑Acetate, ↓Propionate, ↑A:P
42	[56]	enzymatic silage ginger straw	Goat	In vivo	↑DMD, ↑ADFD, ↑NDFD
43	[57]	exogenous non-starch polysaccharidases	Sheep	In vivo	↑DMD, ↑OMD, ↑NDFD, ↑ADFD, ~total VFA, ↑Acetate, ↑Propionate, ↓A:P
44	[58]	fibrolytic enzyme	Dairy cows	In vivo	↑DMI, ↑Milk Production, ↑Lactose, ↑Protein, ↓Fat

DMI: Dry Matter Intake; OMI: Organic Matter Intake; DMD: Dry Matter Digestibility; OMD: Organic Matter Digestibility; CPD: Crude Protein Digestibility; ADFD: Acid Detergent Fibre Digestibility; NDFD: Neutral Detergent Fibre Digestibility; GP: Gas Production in Hour; VFA: Volatile Fatty Acid; ↑: increase; ↓: Decrease; ~: unchanged/not significantly different.

**Table 2:** Meta analysis of the effect of dietary enzymes on in vivo digestibility and gas production in ruminant animals.

		Model Results								Heterogeneity			
Response Variable	N	Unit	Mean Control ± SE	Mean Experiment ± SE	Estimate	Lower Bound	Upper Bound	SE	*p*-Value	τ^2^	Q	Het *p*-Value	I^2^
DMI	17	kg/head/d	25.4 ± 1.56	25.4 ± 1.55	0.110	−0.216	0.435	0.166	0.509	0.049	17.9	0.331	10.5
OMI	6	kg/head/d	23.8 ± 0.77	24.3 ± 0.800	0.229	−0.421	0.880	0.332	0.489	0.121	6.12	0.294	18.3
DMD	41	%	67.3 ± 0.820	67.9 ± 0.870	0.621	0.144	1.10	0.243	0.011	1.827	232	<0.001	83.2
OMD	35	%	68.7 ± 1.00	68.7 ± 1.16	0.521	−0.034	1.09	0.249	0.036	1.644	186	<0.001	81.7
ADFD	28	%	49.3 ± 1.55	51.6 ± 1.87	0.804	0.176	1.43	0.320	0.012	1.963	159	<0.001	83.1
NDFD	38	%	53.8 ± 1.18	56.4 ± 1.39	0.791	0.304	1.28	0.248	0.001	1.662	210	<0.001	82.4
Milk Production	57	kg/day	34.6 ± 1.08	35.5 ± 1.11	0.325	0.159	0.492	0.085	<0.001	0.162	105	<0.001	49.9
Lactose	53	%	4.75 ± 0.019	4.78 ± 0.019	0.142	0.001	0.284	0.072	0.049	0.057	65.9	0.053	25.7
Protein	55	%	3.13 ± 0.043	3.14 ± 0.041	−0.060	−0.189	0.069	0.066	0.362	0.024	57.6	0.245	11.4
Fat	59	%	3.78 ± 0.085	3.74 ± 0.859	−0.103	−0.206	0.001	0.053	0.051	0.000	48.5	0.719	0
Total VFA	29	mM	113 ± 3.41	115 ± 3.38	0.152	−0.065	0.369	0.111	0.170	0.138	51.5	0.001	51.4
Acetate	32	mM	339 ± 49.9	329 ± 48.3	−0.815	−1.357	−0.273	0.277	0.003	1.62	250.9	<0.001	87.6
Propionate	32	mM	124 ± 17.5	134 ± 19.6	0.904	0.331	1.48	0.292	0.002	1.91	279.8	<0.001	88.9
A:P	29	-	2.68 ± 0.130	2.63 ± 0.140	0.232	−0.254	0.717	0.248	0.350	1.39	216.7	<0.001	87.1

DMI: Dry Matter Intake (intensive reared dairy cows); OMI: Organic Matter Intake (intensive reared dairy cows); DMD: Dry Matter Digestibility; OMD: Organic Matter Digestibility; ADFD: Acid Detergent Fibre Digestibility; NDFD: Neutral Detergent Fibre Digestibility; VFA: Volatile Fatty Acid; A:P: Acetate to Propionate ratio; N: sample size per comparison; Unit: measurement unit; Mean control: mean of the control group; Mean experiment: mean of the experiment group; Estimate: pooled effect size; Lower Bound: lower limit of the confidence interval; Upper Bound: upper limit of the confidence interval; SE: standard error of the pooled estimate; *p*-Value: significance level; τ^2^ variability between-study heterogeneity; Q: Cochran’s Q statistic for heterogeneity; Het *p*-value: *p*-value for the heterogeneity test (Q); I^2^: heterogenity (%).

**Table 3:** Meta-analysis of dietary enzymes on the in vitro performance, digestibility, rumen fermentation and milk yield of ruminant animals.

		Model Results								Heterogeneity			
Response Variables	N	Unit	Mean Control ± SE	Mean Experiment ± SE	Estimate	Lower Bound	Upper Bound	SE	*p*-Value	τ^2^	Q	Het *p*-Value	I^2^
DMD	39	%	58.3 ± 2.76	57.0 ± 3.22	0.247	0.024	0.523	0.127	0.032	0.207	60.1	0.013	36.7
OMD	33	%	45.8 ± 3.56	32.8 ± 5.96	2.08	0.318	3.85	0.901	0.021	13.3	505	<0.001	93.7
CPD	6	%	62.1 ± 1.82	70.4 ± 2.27	1.76	−1.73	5.25	1.78	0.322	9.26	39.4	<0.001	87.3
ADFD	15	%	44.8 ± 3.78	49.1 ± 4.20	0.936	0.078	1.79	0.438	0.032	1.54	40.1	<0.001	65.1
NDFD	14	%	45.3 ± 3.18	44.2 ± 3.89	1.410	0.489	2.33	0.470	0.003	2.30	62.6	<0.001	72.8
pH	25	-	6.57 ± 0.066	6.51 ± 0.072	−0.402	−0.816	0.012	0.211	0.057	0.76	91.6	<0.001	73.8
GP 12	15	mL/g DM	51.0 ± 10.5	66.7 ± 8.57	0.854	0.126	1.58	0.371	0.022	1.47	54.5	<0.001	74.3
GP 24	15	mL/g DM	77.6 ± 11.5	103 ± 9.18	0.827	0.284	1.37	0.277	0.003	0.810	50.7	<0.001	72.4
GP 48	15	mL/g DM	114. ± 11.8	150 ± 9.80	0.929	0.536	1.32	0.201	<0.001	0.297	28.1	0.014	50.1
GP 72	15	mL/g DM	138 ± 11.6	176 ± 9.55	0.823	0.427	1.22	0.202	<0.001	0.308	28.9	0.011	51.4

DMD: Dry Matter Digestibility; OMD: Organic Matter Digestibility; ADFD: Acid Detergent Fibre Digestibility; NDFD: Neutral Detergent Fibre Digestibility; GP: Gas Production; N: sample size per comparison; Unit: measurement unit; Mean control: mean of the control group; Mean experiment: mean of the experiment group; Estimate: pooled effect size; Lower Bound: lower limit of the confidence interval; Upper Bound: upper limit of the confidence interval; SE: standard error of the pooled estimate; *p*-Value: significance level; τ^2^ variability between-study heterogeneity; Q: Cochran’s Q statistic for heterogeneity; Het *p*-value: *p*-value for the heterogeneity test (Q); I^2^: Heterogenity (%).

## Data Availability

All data analyzed in this study were obtained from previously published sources, which are fully cited in the reference list. No new experimental data were generated in this study. All relevant information supporting the findings of this meta-analysis is available within the cited publications.

## Abbreviations

The following abbreviations are used in this manuscript:

DMI	Dry Matter Intake
OMI	Organic Matter Intake
DMD	Dry Matter Digestibility
OMD	Organic Matter Digestibility
CPD	Crude Protein Digestibility
ADFD	Acid Detergent Fibre Digestibility
NDFD	Neutral Detergent Fibre Digestibility
GP	Gas Production (hour)
VFA	Volatile Fatty Acid
